# Vaccinations in Paediatric Rheumatology: an Update on Current Developments

**DOI:** 10.1007/s11926-015-0519-y

**Published:** 2015-05-30

**Authors:** Noortje Groot, Marloes W. Heijstek, Nico M. Wulffraat

**Affiliations:** Department of Pediatrics, Wilhemina Children’s Hospital/University Medical Centre Utrecht (UMCU), PO-box 85090, 3508 AB Utrecht, The Netherlands; Department of Pediatric Immunology, Sophia Children’s Hospital/Erasmus Medical Centre, Rotterdam, The Netherlands; Department of Internal Medicine, University Medical Centre Utrecht (UMCU), Utrecht, The Netherlands

**Keywords:** Vaccinations, Paediatric rheumatic diseases, pedRD, Infection prevention, Vaccine immunogenicity, Vaccine safety

## Abstract

In 2011, the European League Against Rheumatism (EULAR) published recommendations regarding the vaccination of children with rheumatic diseases. These recommendations were based on a systematic literature review published in that same year. Since then, the evidence body on this topic has grown substantially. This review provides an update of the systematic literature study of 2011, summarizing all the available evidence on the safety and immunogenicity of vaccination in paediatric patients with rheumatic diseases. The current search yielded 21 articles, in addition to the 27 articles described in the 2011 review. In general, vaccines are immunogenic and safe in this patient population. The effect of immunosuppressive drugs on the immunogenicity of vaccines was not detrimental for glucocorticosteroids and methotrexate. Biologicals could accelerate a waning of antibody levels over time, although most patients were initially protected adequately. Overall, persistence of immunological memory may be reduced in children with rheumatic diseases, which shows the need for (booster) vaccination. This update of the 2011 systematic literature review strengthens the evidence base for the EULAR recommendations, and it must be concluded that vaccinations in patients with rheumatic diseases should be advocated.

## Introduction

Children with paediatric rheumatic diseases (pedRD) have an increased risk of infection, which contributes to the mortality and morbidity of their disease [1–3]. Effective and safe vaccination is key in prevention of numerous of these infections.

Assessing efficacy of a vaccine in patients with pedRDs is challenging. The ideal measure of efficacy, infection rates, is usually not studied as a primary endpoint because this requires large sample sizes. Surrogate measures such as immunogenicity are commonly used instead. Immunogenicity refers to the immune response induced by vaccination. This is usually measured by vaccine-specific geometric mean antibody titers (GMT) or concentrations (GMC), seroconversion rates and/or seroprotection rates. The measure for immunogenicity differs per vaccine, as the relation between the humoral and/or cellular immune response and protection differs per pathogen [4–6]. Immunogenicity of a vaccine in patients with rheumatic diseases can differ from the healthy population, due to the disease or its immunosuppressive treatment.

Besides short-term vaccine-induced immune responses, *persistence* of protective immunologic memory after vaccination is essential in preventing infections [7, 8]. As this persistence goes beyond follow-up of most studies in rheumatic diseases, long-term effectiveness of most vaccines is unknown.

The safety of vaccines in pedRD can be addressed on different levels: adverse event rate in comparison to healthy controls, increased disease activity induced by vaccination and unintentional infections induced by live-attenuated pathogens in vaccines (especially in patients on high-dose immunosuppressive drugs). Another issue of vaccine safety is whether vaccines or their constituents can actually cause autoimmune disease (AID), which will be addressed briefly.

Over the years, awareness of infection prevention by vaccination in rheumatic diseases has increased. In 2011, a EULAR task force published evidence-based recommendations regarding vaccination of adults and children with rheumatic diseases. A year later, the Brazilian Society of Rheumatology published vaccination recommendations for patients with rheumatoid arthritis (RA) [9, 10, 11••].

According to these recommendations, non-live vaccines are generally adequately immunogenic and safe. Live-attenuated vaccines can be administered to patients with pedRD, unless they are on high-dose immunosuppressive drugs or biologicals. In these cases, evidence on safety is scarce but reassuring. Therefore, live-attenuated *booster* vaccinations can be considered on individual basis.

Not all vaccines have been studied in pedRD patients, most studies do not take persistence of immunological memory into account, and studies were often underpowered and uncontrolled to assess safety. Consequently, concerns regarding efficacy and safety of vaccines remain. Providing a periodical overview of new evidence, as advised in the EULAR recommendations, is necessary to assure effective and safe vaccination in this vulnerable group.

In this review, we provide an update of the evidence on vaccination of pedRD patients published since the EULAR recommendations in 2011 [12••]. The influence of immunosuppressive drugs and biologicals on immunogenicity and safety of non-live composite as well as live-attenuated vaccines will be addressed. Additionally, we describe the use of adjuvants and their possible association with adverse events (AE).

**Fig. 1:**
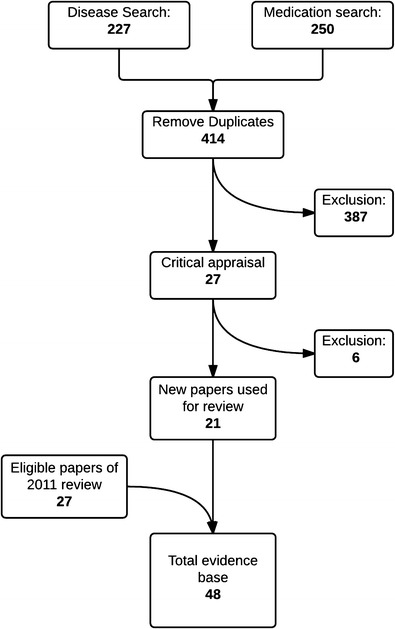
The search strategy for the systematic literature review [12••]. The disease search encompassed articles on vaccination in patients with paediatric autoinflammatory or rheumatic diseases, and the medication search encompassed articles on vaccination and immunosuppressive drugs

A systematic literature review was performed in July 2014, following the methodology described earlier [12••]. Since the first systematic literature review describing 27 papers, 21 additional eligible articles on vaccination of patients with pedRD have been published (Fig. 1). A large portion (*n* = 10) of the new studies investigated the immunogenicity of the seasonal influenza or H1N1 vaccine. The pedRD studied most (*n* = 13) was juvenile idiopathic arthritis (JIA). Eleven new studies described the influence of biologicals on immunogenicity of the vaccine, adding to the five studies described earlier. Three additional articles were found which studied live-attenuated vaccines to the six articles included in 2011. Two new studies were randomized controlled trials (RCTs). This is the study design of choice when assessing the effect of vaccination on disease activity in pedRD [13••, 14••].

## Vaccine Immunogenicity in Paediatric Patients With Rheumatic Diseases

Most studies assessed short-term vaccine-induced immunogenicity. Five studies measured antibody levels up to 12 months post-vaccination [13••, 14••, 15–17]. Another five studies evaluated antibody persistence several years after vaccination [18–22]. Although some studies studied actual occurrence of infections such as herpes zoster (HZ) or influenza, they were underpowered to assess these outcomes reliably [13••, 23, 24].

Below we summarize and discuss all available evidence found in the previous and current systematic literature [12••].

### Immunogenicity in Relation to Immunosuppressive Drugs

#### Glucocorticosteroids

Eleven articles included 401 patients using glucocorticosteroids (GC). Of them, the majority used a low dose (<20 mg/day) [17, 18, 25–27, 29–32, 43] (Table 1). Patients who use GC may show lower seroconversion rates or GMT, but they generally still reach protective antibody titers. A high dose of GC or concomitant use of other immunosuppressive drugs was associated with lower, yet still protective, responses in several studies. No effect of GC on the persistence of several vaccine-specific antibodies (mumps, measles and rubella vaccine (MMR), tetanus-diphtheria vaccine (TD)) could be found in one study [18]. These findings show that there is no general detrimental effect of low-dose GC on immunogenicity or established antibody levels.

**Table 1:** Critical appraisal of available evidence on effects of immunosuppressive drugs on immunogenicity and safety of vaccines in PedRD

Reference	Medication	Vaccine	LoE	Immunogenicity	Safety
Glucocorticosteroids					
Kanakoudi-Tsakalidou et al. [25]	16 GC ≤0.5 mg/kg/day 16 GC ≤0.5 mg/kg/day + MTX 15–20 mg/m^2^/week 11 GC ≤0.5 mg/kg/day + CY 2.5–3.5 mg/kg/day 6 GC ≤0.5 mg/kg/day + AZA 2–2.5 mg/kg/day 8 GC ≤0.5 mg/kg/day + MTX + CY *Versus 13 ARD without GC* (*5 MTX*, *4 CY*, *4 MTX* + *CY*) *and 5 HC*	Influenza	3	No effect of GC on antibody concentrations or response rate compared to patients without GC. No flu-like symptoms in any of the patients until 6 months after vaccination.	–
Kasapçopur et al. [26]	20 GC 6.05 mg (2.5–10 mg/day) *Versus 19 JIA without GC and 41 HC*	HBV	3	No effect of GC on antibody concentration (GMT 109.7 IU/ml versus 141.1 IU/ml) or response rate.	No increase in disease activity.
Kiray et al. [27]	55 GC 2.5–40 mg/day *Versus 60 JIA without GC*	BCG	3	No effect of GC on PPD induration size (3.9 mm versus 4.7 mm) several years after BCG vaccination. PPD positivity rate similar in GC users and nonusers	–
Lu et al. [28]	12 GC <2 mg/kg/day Versus *19 non-immunosuppressed IBD*	Influenza	3	No effect of GC on seroprotection rate against 3 influenza strains. Higher post vaccination GMT for strain B compared to patients without IS drugs.	No difference in adverse events and disease activity compared to patients without IS drugs.
Pileggi et al. [17]	13 GC 0.1–0.7 mg/kg/day All combined with MTX 12–25 mg/m^2^/week 3 combined with CY 3–3.5 mg/kg/day 1 combined with leflunomide 10 mg/day 1 combined with penicillamine 13 mg/kg/day *Versus 12 ARD without GC*	VZV	3	Seroprotection 8/13 patients on GC versus 7/12 patients on MTX monotherapy.	2/13 patients with GC + MTX + DMARD had mild self-limiting VZV-like rash, compared to 1/12 patient with MTX monotherapy. No increase in disease activity, no increase in medication use.
Miyamoto et al. [20]	12 GC 0.2–0.9 mg/kg/day post vaccination *Versus 7 SLE without GCs post vaccination*	MMR DTP	3	No effect of GC on established antibody concentrations or seroprotection rate.	–
Ogimi et al. [29]	14 GC 0.18 ± 0.17 mg/kg *Versus 36 HC*	Influenza	3	Similar anti-influenza antibody concentrations and seroconversion rate as HC.	–
Aytac et al. [30]	17 GC mean dose 6.25 mg/day 11 combined with AZA mean dose 100 mg/day 3 combined with MMF mean dose 1000 mg/day 2 combined with HCQ mean dose 200 mg/day 3 without medication *Versus 24 HC*	HBV	3	GMT not significantly lowered by GC or AZA; however, large proportion of patients used these medications and a control group was lacking. Not-significant negative correlation between prednisone use and anti-HBs titres. Differences in seroconversion or seroprotection were not reported.	–
Heijstek et al. [18]	23 oral GC <20 mg/day median dose 10 mg/day) 5 oral GC ≥20 mg/day 246 NSAID 93 MTX median dose 10 mg/m^2^/week 24 DMARD 8 anti-TNFα *Versus 2176 HC*	MMR	3	No effect of GC at time of sampling on level of antibodies or seroprotection rates several years after vaccination.	–
Aikawa et al. [31]^a^	54 GC <20 mg/day 36 GC ≥ 20 mg/day 74 MTX 43 AZA 23 cyclosporin 13 MMF 6 leflunomide 3 CY *Versus 92 HC*	Influenza	3	No differences in the seroconversion rate, seroprotection rate were seen between treatment groups. Lower GMT were seen in patients who used AZA, MMF or GC, especially in a dose >20 mg, or patients who used GC and IS.	–
Campos et al. [32]	92 Antimalarials 43 GC <20 mg/day 40 GC ≥20 mg/day 44 AZA 15 MMF 14 MTX 3 CYC 2 CSP *Versus 102 HC*	Influenza	3	Non-seroconversion was associated with a higher prednisone dose in univariate analysis. No effect of medication in multivariate analysis.	–
Methotrexate					
Kasapçopur et al. [26]	22 MTX 10 mg/m^2^/week *Versus 17 without MTX and 41 HC*	HBV	3	No effect of MTX on antibody concentration (GMT 114.4 versus 137 IU/ml) or response rate.	No increase in disease activity.
Heijstek et al. [33]	49 MTX 7–25 mg/m^2^/week *Versus 158 JIA without MTX*	MMR	3	–	No increase in disease activity.
Kiray et al. [27]	73 MTX 3–20 mg/week *Versus 42 JIA without MTX*	BCG	3	No effect of MTX on PPD induration size (4.3 versus 3.9 mm) several years after BCG vaccination. PPD positivity rate similar in MTX users and nonusers.	–
Borte et al. [34]	5 MTX 10 mg/m^2^/week 5 MTX 10 mg/m^2^/week + anti-TNFα 0.4 mg/kg *Versus 22 HC and 5 JIA with MTX 10 mg*/*m2*/*week 4 years post MMR*	MMR	3	No effect of MTX during vaccination on cellular or humoral immunity.	No increase in disease activity or medication use after MMR booster, irrespective of MTX use. No overt measles, mumps or rubella infection induced by vaccination.
Woerner et al. [35]	18 MTX 10 anti-TNFα 8 MTX + anti-TNFα 7 GC <0.5 mg/kg combined with MTX or anti-TNFα *Versus 16 HC*	Influenza	2b	No significant difference in seroconversion, seroprotection or GMT between treatment groups. No effect of MTX on relative difference between pre- and post vaccination GMT in multivariate analysis.	–
Aikawa et al. [36]^a^	47 MTX 5–50 mg/week 63 DMARD/IS (prednisone, leflunomide, CYC, SSZ) 16 anti-TNFα *Versus 91 HC*	Influenza	3	Seroconverted patients (83.2 %) and non-seroconverted patients (16.8 %) had similar types of therapy and doses of each therapy.	–
Heijstek et al. [18]	93 MTX median dose 10 mg/m^2^/week 23 oral GC <20 mg/day median dose 10 mg/day) 5 oral GC ≥20 mg/day 246 NSAID 24 DMARD 8 anti-TNFα *Versus 2176 HC*	MMR	3	No effect of MTX at time of sampling on level of antibodies or seroprotection rates several years after vaccination.	–
Stoof et al. [22]	108 MTX 60 biologicals 14 GC *Versus 1527 HC*	MenC	3	No effect of MTX on decline of antibody levels over time.	–
IVIG					
Tacke et al. [37]	150 IVIG *Versus 92 HC*	MMR	3	Seroprotection and GMT reduced until 9 months after IVIG treatment	–
Biologicals					
Borte et al. [34]	5 MTX 10 mg/m^2^/week + anti-TNFα 0.4 mg/kg *Versus 22 HC and 10 JIA without anti-TNFα*	MMR	3	No effect of anti-TNFα during vaccination on cellular or humoral immunity.	No increase in disease activity or medication use after MMR booster, irrespective of anti-TNFα. No overt measles, mumps or rubella infection induced by vaccination.
Lu et al. [28]	45 anti-TNFα *Versus 19 non-immunosuppressed IBD*	Influenza	3	Lower response rate to strain B in patients on anti-TNFα (14 %) compared with patients without IS drugs (39 %).	No difference in adverse events and disease activity compared to patients without IS drugs.
Lu et al. [38]	2 anti-TNFα (infliximab) + 6-MP *Versus 4 IBD with 6-MP monotherapy* (*1.5*–*2 mg*/*kg*/*day*)	VZV	4	No effect of anti-TNFα during vaccination on seroprotection rate (100 %). Proper control group is lacking.	No serious adverse events after primary/booster VZV vaccination, despite anti-TNFα usage.
Farmaki et al. [39]	31 MTX/CY ± GC + anti-TNFα *Versus 32 MTX*/*CY* ± *GC*	PCV7	2B	Lower antibody concentrations against 3/7 serotypes in patients on anti-TNFα, but similar response and protection rate.	Mild adverse events in 6/31 patients on anti-TNFα versus 5/32 patients without anti-TNFα.
Erguven et al. [40]	4 anti-TNFα *Versu*s *43 JIA without anti-TNFα but with GC and*/*or DMARD and 67 HC*	HAV	3	4 patients on anti-TNFα negative for anti-HAV antibodies after vaccination. 100 % response rate in all other patients and HC.	No adverse events. No increase in disease activity.
Woerner et al. [35]	10 anti-TNFα 8 MTX + anti-TNFα 18 MTX 7 GC <0.5 mg/kg combined with MTX or anti-TNFα *Versus 16 HC*	Influenza	3	No significant difference in seroconversion, seroprotection or GMT between treatment groups. Analysis of the effect of biologicals on relative difference between pre- and post vaccination GMT in multivariate analysis showed a trend towards a lower relative change.	–
Dell’Era et al. [41]	30 DMARD (unspecified) 18 DMARD + MTX mean dose 10 ± 1.4 mg/m^2^/week 30 anti-TNFα 14 anti-TNFα + MTX 10 ± 1.4 mg/m^2^/week *Versus 30 HC*	Influenza	3	Patients using anti-TNFα had significantly lower seroconversion rates and seroprotection rates against strain B, a significantly lower GMT against H1N1 and B, and showed a more rapid decline of GMT over time.	–
Toplak et al. [24]	7 DMARD + GC (<10 mg/day) 4 anti-TNFα 3 leflunomide 2 sulphasalazine *Versus 18 patients without therapy and 14 HC*	Influenza	2b	All patients on anti-TNFα were seroprotected, but they had a smaller increase in GMT after vaccination.	–
Aikawa et al. [36]^a^	16 anti-TNFα 63 DMARD/IS (prednisone, leflunomide, CYC, SSZ) 47 MTX 5–50 mg/week *Versus 91 HC*	Influenza	IG: 2b DAS: 3	Seroconverted patients (83.2 %) and non-seroconverted patients (16.8 %) had similar types and doses of therapy.	–
Carvalho et al. [23]	31 MTX or leflunomide 6 GC dose 0.05–1 mg/kg/day 5 anti-TNFα 1 CSP *Versus 10 HC*	Influenza	3	Anti-TNFα users had lower seroconversion and seroprotection rates to the H1N1 strain (60 versus 100 % in HC). 80 % of patients were seroprotected against the H2N3 and B strains, compared to 80 % and 100 % of HC, respectively.	–
Moses et.al. [21]	78 anti-TNFα mean dose 6.9 ± 1.8 mg/kg 53 AZA 36 MTX 14 6-MP	HBV	3	56 % of the 87 patients were still seroprotected after HBV vaccination in the past, and 76 % of the 34 patients who received the booster vaccine were seroprotected 1 month after administration.	–
Heijstek et al. [15]	37 NSAIDs 24 MTX mean dose 10.2 mg/m^2^/week 9 anti-TNFα median dose 45 mg/week 6 other DMARDs 1 anti IL-1R *Versus 55 HC*	bHPV	3	All patients using anti-TNFα were seropositive after 3 vaccines, with lower GMTs.	–
Stoof et al. [22]	108 MTX 60 biologicals 14 GC *Versus 1527 HC*	MenC	3	Use of biologicals accelerated the decline of antibody levels over time.	–
Heijstek et al. [14••]	Vaccinated: 38 NSAIDs 29 MTX mean dose 10.6 mg/m^2^/week 6 anti-TNFα median dose 15 mg/week 3 anti IL-1R median dose 1.6 mg/kg 2 oral GC 1 leflunomide Unvaccinated: 36 NSAIDs 31 MTX mean dose 11.6 mg/m^2^/week 4 anti-TNFα median dose 21 mg/week 2 anti IL-1R median dose 1.4 mg/kg 1 oral GC 1 leflunomide	MMR	3	All patients using biologicals were seroprotected against measles, rubella and mumps.	No MMR infections induced by vaccine in patients on DMARDs or in patients on biologicals.
Shinoki et al. [42]	27 anti-IL6 24 GC (mean dose 7.3 mg/day) *Versus 17 HC*	Influenza	3	Seroconversion, seroprotection and GMTs similar in patients using anti-IL6 and healthy controls	–

Adapted from Heijstek et al. Vaccination in paediatric patients with auto-immune rheumatic diseases: a systemic literature review for the European League against rheumatism evidence-based recommendations, Autoimmunity reviews 2011;11;112–122

*AIH* auto-immune hepatitis patient, *ARD* auto-immune rheumatic disease, *AZA* azathioprine, *BCG* Bacillus Calmette-Guérin, *CFM* cyclophosphamide, *CY* cyclosporine A, *DMARD* disease-modifying anti-rheumatic drug, *GC* glucocorticosteroids, *GMC* geometric mean concentration, *GMT* geometric mean titres, *HAV* hepatitis A virus, *HBV* hepatitis B virus, *HC* healthy controls, *HCQ* hydroxychloroquine, *HPV* human papillomavirus, *IBD* inflammatory bowel disease patient, *IL6* interleukin-6, *IS* immunosuppressive, *ITP* idiopathic thrombocytopenic purpura patient, *JDM* juvenile dermatomyositis patient, *JIA* juvenile idiopathic arthritis patient, *JScl* juvenile scleroderma patient, *JSLE* juvenile systemic lupus erythematosus patient, *KD* Kawasaki disease patient, *LoE* level of evidence, *6-M* 6-mercaptopurine, *MenC* meningococcal serogroup C conjugate vaccine, *MCTD* mixed connective tissue disease patient, *MMF* mycophenolate mofetil, *MMR* measles, mumps, rubella, *MTX* methotrexate, *NSAID* non-steroid anti-inflammatory drugs, *NVP* national vaccination programme, *OR* odds ratio, *PCV7* 7-valent pneumococcal conjugate vaccine, *pedRD* paediatric rheumatic diseases, *pIBD* paediatric inflammatory bowel disease patient, *PPD* purified protein derivative of tuberculin, *RMO* recurrent multifocal osteomyelitis patient, *soJIA* systemic onset juvenile idiopathic arthritis patient, *TD* tetanus-diphtheria vaccine, *TNFα* tumour necrosis factor alpha, *TT* tetanus toxoid, *VZV* varicella zoster virus

^a^These studies overlapped in patient population

#### Methotrexate

Eight studies including 420 patients on methotrexate (MTX) were available [18, 20, 26, 27, 33–36] (Table 1). No effect of MTX was found on short-term immunogenicity of vaccines or on the persistence of antibodies over time [18, 22].

#### Biologicals

A total of 296 patients using biologicals were included in 15 studies [13••, 14••, 21–24, 28, 34–41] (Table 1). The biologicals most frequently studied were tumour necrosis factor (TNF)α blockers. The majority of patients reached protective antibody concentrations after vaccination, but in the majority of studies the actual antibody concentrations of patients using biologicals were lower than of patients who did not. Additionally, the antibody levels declined more rapidly over time in patients using biologicals [22, 41]. A lower initial GMT and a more rapid decline in antibody levels will lead to a quicker decrease in seroprotection rate in these patients. Monitoring GMTs and additional booster vaccinations should be considered in order to ensure protection in these patients. Another option is to administer specific vaccines prior to start of biological therapy.

### Immunogenicity of Non-live Composite Vaccines

#### Human Papillomavirus Vaccine

Currently, there are two human papilloma virus (HPV) vaccines: the quadrivalent (qHPV) vaccine (against HPV 6, 11, 16 and 18) and the bivalent (bHPV) vaccine (against HPV 16 and 18). At the time the EULAR recommendations were published, no publications regarding the immunogenicity or safety of either HPV vaccine in pedRD were available. The recommendation was based on preliminary data [10]. Since then, three articles assessing the immunogenicity and safety of the HPV vaccine in pedRD have been published [15, 16, 52] (Table 2).

**Table 2:** Critical appraisal of available evidence on immunogenicity and safety of vaccines in pedRD

Vaccine	Patients	Medication	LoE	Immunogenicity	Safety
Live-attenuated					
Bacillus Calmette-Guérin					
Hsu et al. [44]	281 KD	Unknown	3	–	Local inflammation at BCG vaccination site in up to 50 % of KD patients.
Kuniyuki et al. [45]	1 KD	Unknown	4	–	Case report of local inflammation at BCG vaccination site.
Antony et al. [46]	2 KD	Unknown	4	–	Case report of local inflammation at BCG vaccination site.
Weinstein [47]	1 KD	Unknown	4	–	Case report of local inflammation at BCG vaccination site.
Chalmers et al. [48]	1 KD	Unknown	4	–	Case report of local inflammation at BCG vaccination site.
Kiray et al. [27]	115 JIA 45 HC	55 GC 73 MTX 17 sulphasalazine	2B	PPD reactivity several years after 1–2 BCG vaccinations: induration size smaller in JIA patients, 39 % JIA versus 84 % HC reacted to PPD. No influence of IS drugs.	–
Uehara et al. [49]	15,524 KD	Unknown	3	–	Local inflammation at BCG vaccination site in 50 % of KD patients.
Measles, mumps, rubella					
Drachtman et al. [50]	1 ITP	None	4	–	Case report of a flare of ITP 7 weeks after MMR booster.
Heijstek et al. [27]	207 JIA	49 MTX	2B	–	No increase in disease activity.
Borte et al. [34]	15 JIA 22 HC	5 MTX 4 years post MMR 5 MTX 5 MTX + anti-TNFα	2B	No interference of MTX or anti-TNFα with cellular or humoral immunity.	No increase in disease activity or medication use after MMR booster. No influence of MTX or anti-TNFα.
Korematsu et al. [51]	1 JIA	NSAIDS	4	–	Case report of a flare of systemic JIA 5 days after rubella vaccination.
Miyamoto et al. [20]	30 JSLE 14 HC	25 HCQ 19 oral GC 14 AZA 9 IV GC 2 CFMpulse 2 CY 2 MTX 1 MMF	2B	At 7–16 years after vaccination, protective antibody levels against measles were similar in patients and controls.	–
Heijstek et al. [18]	400 JIA 2176 HC	246 NSAID 93 MTX 28 oral GC (median dose 10 mg/day) 24 DMARD 8 anti-TNFα	2C	Protective antibody levels against mumps and rubella in patients were lower after past vaccination (time since vaccination up to 10 years; adjusted OR for seroprotection between 0.1 and 0.4). Protective antibody levels against measles did not significantly differ from controls.	-
Heijstek et al. [14••]	68 JIA patients (vaccinated) 69 JIA patients, (unvaccinated)	Vaccinated: 38 NSAIDs 29 MTX 6 anti-TNFα 3 anti-IL1-R 2 oral GC 1 leflunomide Unvaccinated: 36 NSAIDs 31 MTX 4 anti-TNFα 2 anti-IL1-R 1 oral GC 1 leflunomide	1B	All vaccinated patients had protective antibody levels against MMR, with a significant increase in GMC. Two patients became seronegative over time.	No MMR infections induced by vaccine. Frequency of flares was similar in vaccinated and unvaccinated patients. Patients on biologics did not show any MMR infections, flares or increase in disease activity.
Varicella zoster virus					
Pileggi et al. [17]	17 JIA 4 JDM 4 other ARD 18 HC	13 GC 4.2 mg/day 25 MTX 5 DMARD	2B	Seroprotection 50 % in patients versus 72 % in HC (within range of historical healthy cohort). 2 of 8 patients that were exposed to VZV developed chickenpox, 1 of these patients was on anti-TNFα.	3 patients with mild self-limiting VZV-like rash. No increase in disease activity.
Lu et al. [38]	6 IBD	6 6-MP 2 anti-TNFα	4	Seroprotection in 5/6 patients shortly after VZV vaccination.	No serious adverse events after primary/booster VZV vaccination, despite anti-TNFα usage.
Barbosa et al. [13••]	28 JSLE patients (vaccinated) 26 JSLE patients (unvaccinated) 28 HC	Vaccinated: 27 HCQ 18 GC (mean dose 7.5 ± 3.9 mg) 9 AZA 2 MTX Unvaccinated: 22 HCQ 18 GC (mean dose 9.4 ± 4.8) 12 AZA 2 CFM	1B	Patients showed a similar increase in GMT as healthy controls. *NB*: *seroprotection 100* % *in patients and controls before vaccination*.	Frequency of flares was similar in vaccinated and unvaccinated patients.
Non-live composite					
Human papilloma virus					
Soybilgic et al. [52]	27 JSLE	27 HCQ 16 GC (mean dose 12.6 mg) 9 AZA 9 MMF 6 MTX	3	All but one patient seroconverted for all 4 HPV types.	No increase in disease activity after vaccination.
Heijstek et.al. [16]	6 JSLE 6 JDM 49 HC	6 GC 2 HCQ 2 MTX 1 AZA 1 MMF	2b	All but one JDM patient and all controls seroconverted after the third dose. The GMT in patients was lower than in HC.	No increase in disease activity after vaccination.
Heijstek et al. [15]	68 JIA 55 HC	37 NSAIDs 24 MTX 9 anti-TNFα 6 other DMARDs 1 anti-IL-1R	2b	All participants were seropositive after vaccination. The GMT in patients was lower than in HC.	No disease flares, no increase in disease activity after vaccination.
Hepatitis A virus					
Beran et al. [53]	10 AIH	Unknown	3	100 % response rate.	No severe adverse events. No increase in disease activity.
Erguven et al. [40]	47 JIA 67 HC	12 GC 29 MTX 11 GC + MTX 19 sulphasalazine 4 anti-TNFα	2B	4 patients on anti-TNFα (systemic JIA) negative for anti-HAV antibodies after vaccination. 100 % response rate in all other patients and HC.	No adverse events. No increase in disease activity.
Moses et al. [54]	12 pIBD	12 anti-TNFα 2 MTX	3	Seroconversion rate was 92 %.	–
Hepatitis B virus (DNA)					
Kasapçopur et al. [26]	39 JIA 41 HC	20 GC 22 MTX	2B	Seroprotection in 38/39 patients vaccination, comparable to HC. No effect IS drugs.	No increase in disease activity.
Beran et al. [53]	10 AIH	Unknown	3	100 % response rate in patients <15 years. 50 % response rate in 4 patients aged 16–20 year, 1 used GCs 5 mg/day.	No severe adverse events. No increase in disease activity.
Aytac et al. [30]	20 JSLE 24 HC	17 GC (mean dose 6.25 mg/day) 11 AZA 3 MMF 2 HCQ 3 no medication	2B	Seroconversion and seroprotection lower in patients than in controls (80 versus 100 %). The GMT in patients was lower than in HC.	No increase in disease activity after vaccination.
Moses et al. [21]	87 pIBD, of whom 34 received booster vaccine	87 anti-TNFα (mean 6.9 ± 1.8 mg/kg/dose) 53 AZA 36 MTX 14 6-MP	3	56 % of patients were protected after HBV vaccination in the past; 76 % of 34 patients had an adequate response to the booster vaccine.	–
Maritsi et al. [19]	89 newly diagnosed JIA 89 HC	None: study measured protective antibody levels from NVP	2B	After a median time after vaccination of 5 years, the level of protective anti-HBs-antibody levels was significantly lower in JIA patients (55 %) than in HC (92 %).	–
Seasonal influenza					
Denman et al. [55]	3 JIA 20 HC	3 chlorambucil	2B	Similar anti-influenza antibody concentrations. No effect of IS drugs.	–
Malleson et al. [43]	34 JIA 13 HC	7 GC 9 DMARD	2B	Similar anti-influenza antibody concentrations and seroconversion rate as HC. No effect of IS drugs.	Similar adverse events as healthy controls. 4 flares per 145 patient months before versus 3 flares per 34 patient months after vaccination. As a group, more patients improved than deteriorated.
Kanakoudi-Tsakalidou et al. [25]	49 JIA 11 SLE 3 JDM 7 other ARD 5 HC	16 GC 16 GC + MTX 11 GC + CY 6 GC + AZA 8 GC + MTX + CY 5 MTX 4 CY 4 MTX + CY	2B	15 non-responders among patients. Similar immunogenicity between patients.	No severe adverse events. No increase in disease activity.
Mamula et al. [56]	51 IBD 29 HC	12 GC 1 MTX 18 6-MP 10 6-MP + anti-TNFα 6 MTX + anti-TNFα	2B	In general, lower responses to 1 strain compared with HC. Lower responses in patients on anti-TNFα + DMARDs towards 2 strains.	Similar non-severe adverse events as HC. No increase in disease activity.
Lu et al. [28]	146 IBD	12 GC 59 MTX/AZA/6-MP 45 anti-TNFα 10 tacrolimus	3	In general good immunogenicity. Patients on anti-TNFα lower responses to 1 strain in contrast to other IS drugs.	No severe adverse events. No increase in disease activity.
Ogimi et al. [29]	23 JIA 12 SLE 6 JDM 2 KD 2 MCTD 4 other ARD 36 HC	14 GC 7 GC + MTX 7 GC + MMF 4 GC + other DMARD 13 GC + 2 DMARD 2 CY 1 MTX 1 MTX + CFM + AZA	2B	Similar anti-influenza antibody concentrations and seroconversion rate as HC. No effect of IS drugs. Of note, pre-vaccination anti-influenza antibody concentrations were higher in patients.	Similar non-severe adverse events as HC. 2 patients (1 JIA, 1 Takayasu arteritis) experienced a flare of disease within 2 weeks after vaccination.
Woerner et al. [35]	25 JIA 3 uveitis 2 IBD 2 RMO 1 vasculitis 1 JSLE 1 MCTD 16 HC	18 MTX 10 anti-TNFα 8 MTX + anti-TNFα	2B	Seroprotection and seroconversion were similar in patients and controls. The GMT in patients was lower than in HC.	–
Dell’Era et al. [41]	60 JIA 30 HC	30 DMARD (unspecified) 32 MTX 30 anti-TNFα	2B	Seroprotection and seroconversion were similar in patients treated with DMARDs and controls.	No increase in disease activity after vaccination.
Shimizu et al. [57]	1 soJIA	1 anti-IL6	3	–	Case report of disease flare after influenza vaccination.
Shinoki et al. [42]	27 soJIA 17 HC	27 anti-IL6 24 GC (mean dose 7.3 mg/day)	2B	Seroconversion, seroconversion and GMT were similar to healthy controls.	No increase in disease activity after vaccination.
Toplak et al. [24]	31 JIA (vaccinated) 31 JIA (unvaccinated) 17 HC	18 without therapy 7 DMARD + GC (<10 mg/day) 4 anti-TNFα 3 leflunomide 2 sulphasalazine	2B	Seroprotection similar to controls after 1 month, similar decline in protective antibodies after 6 months.	Flare rate in vaccinated group 36 %, in unvaccinated group 23 %, but the unvaccinated group had less active disease and selection of control group unclear.
Aikawa et al. [31]^a^	99 JSLE 93 JIA 18 JDM 11 JScl 16 vasculitis 91 HC	54 GC <20 mg/day 36 GC ≥20 mg/day 74 MTX 43 AZA 23 CY 13 MMF 6 leflunomide 3 CFM	2B	Compared to HC, seroconversion, seroprotection and GMT were significantly lower in JSLE patients and lower in other pedRD.	–
Guissa et al. [58]^a^	30 JDM 81 HC	12 GC <20 mg/day 3 GC ≥20 mg/day 14 MTX 7 HCQ 6 CY 2 AZA	2B	Similar seroprotection rate in patients and controls. *NB*: *12 of these patients are also included in study of Aikawa* et al. *2012*.	No disease flares, no increase in disease activity after vaccination.
Aikawa et al. [36]^a^	95 JIA 91 HC	63 DMARD/IS (prednisone, leflunomide, CFM sulphasalazine) 47 MTX 16 anti-TNFα	2B	Significantly lower seroconversion in patients, similar seroprotection and GMT in patients and controls. *NB*: *patients in this study are also included in study of Aikawa* et al. *2012*.	No disease flares, no increase in disease activity after vaccination.
Campos et al. [32]	110 JSLE 102 HC	92 antimalarials 43 GC <20 mg/day 40 GC ≥20 mg/day 44 AZA 15 MMF 14 MTX 3 CFM 2 CY	2B	Seroconversion, seroprotection and GMT were significantly lower in patients than in controls. A SLEDAI >8 was associated with non-response in multivariate analysis.	No increase in disease activity after vaccination.
Carvalho et al. [23]	44 JIA 10 HC	31 MTX or leflunomide 6 GC (mean dose 0.3 mg/kg/day) 5 anti-TNFα 1 CY	2B	Seroprotection in patients similar to controls.	No increase in disease activity after vaccination.
Meningococcal (MenC)					
Zonneveld-Huijssoon et al. [59]	234 JIA	36 MTX <10 mg/m^2^/week 15 MTX >10 mg/m^2^/week 7 sulphasalazine 8 anti-TNFα 1 CFM 2 MTX + sulphasalazine	2B	In general, good protection in all JIA patients. Lower MenC-specific antibody responses in patients receiving IS drugs, but sufficient bactericidal activity as patients with high responses towards the MenC vaccination.	No increase in disease activity, no increased risk of a relapse after vaccination.
Stoof et al. [22]	127 JIA 1527 HC	108 MTX 60 biologicals 14 GC	2C	Highest post-vaccination antibody concentrations were seen in the eldest patients at time of vaccination. Antibody levels waned over time in all patients. The persistence over time was similar to healthy controls.	–
Pneumococcal (PCV7)					
Farmaki et al. [39]	63 JIA	32 DMARD ± C 31 DMARD + anti-TNFα ± GC	2B	Lower antibody concentrations against 3/7 serotypes, but similar response and protection rate. No pneumococcal disease or respiratory tract symptoms during 2-year follow-up	No increase in disease activity. Similar mild adverse events in patients with and without anti-TNFα.
Tetanus-diphtheria					
Denman et al. (TT vaccine) [55]	3 JIA 20 HC	3 chlorambucil	2B	Similar anti-TT antibody concentrations. No effect of IS drugs.	–
Höyeraal et al. [60]	34 JIA 34 HC	Unknown	3	Higher antibody humoral responses to TT and diphtheria, although not corrected for higher baseline antibody levels.	–
Kashef et al. (TT vaccine) [61]	40 SLE 60 HC	10 GC + CFM 13 GC + AZA 5 GC + CFM + AZA 8 GC + MMF	3	Several years after vaccination, similar seroprotection rate (100 %) against TT. Influence IS drug unknown.	–
Miyamoto et al. (TT vaccine) [20]	30 JSLE 14 HC	25 HCQ 19 oral GC 14 AZA 9 I.V. GC 2 CFMpulse 2 CY 2 MTX 1 MMF	2B	Patients had protective antibody levels of tetanus antibodies than controls. No effect of IS drugs.	–
Heijstek et al. (TD vaccine) [18]	400 JIA 2176 HC	246 NSAID 93 MTX 28 oral GC (median dose 10 mg/day) 24 DMARD 8 anti-TNFα	2C	Protective antibody levels against diphtheria and tetanus in patients were lower after past vaccination (time since vaccination up to 10 years; adjusted OR for seroprotection between 0.1 and 0.4).	

Adapted from Heijstek et al. Vaccination in paediatric patients with auto-immune rheumatic diseases: a systemic literature review for the European League against Rheumatism evidence-based recommendations, Autoimmunity reviews 2011;11;112–122

*AIH* auto-immune hepatitis patient, *ARD* auto-immune rheumatic disease, *AZA* azathioprine, *BCG* Bacillus Calmette-Guérin, *CFM* cyclophosphamide, *CY* cyclosporine A, *DMARD* disease-modifying anti-rheumatic drug, *GC* glucocorticosteroids, *GMC* geometric mean concentration, *GMT* geometric mean titres, *HAV* hepatitis A virus, *HBV* hepatitis B virus, *HC* healthy controls, *HCQ* hydroxychloroquine, *HPV* human papillomavirus, *IBD* inflammatory bowel disease patient, *IL6* interleukin-6, *IS* immunosuppressive, *ITP* idiopathic thrombocytopenic purpura patient, *JDM* juvenile dermatomyositis patient, *JIA* juvenile idiopathic arthritis patient, *JScl* juvenile scleroderma patient, *JSLE* juvenile systemic lupus erythematosus patient, *KD* Kawasaki disease patient, *LoE* level of evidence, *6-M* 6-mercaptopurine, *MenC* meningococcal serogroup C conjugate vaccine, *MCTD* mixed connective tissue disease patient, *MMF* mycophenolate mofetil, *MMR* measles, mumps, rubella, *MTX* methotrexate, *NSAID* non-steroid anti-inflammatory drugs, *NVP* national vaccination programme, *OR* odds ratio, *PCV7* 7-valent pneumococcal conjugate vaccine, *pedRD* paediatric rheumatic diseases, *pIBD* paediatric inflammatory bowel disease patient, *PPD* purified protein derivative of tuberculin, *RMO* recurrent multifocal osteomyelitis patient, *soJIA* systemic onset juvenile idiopathic arthritis patient, *TD* tetanus-diphtheria vaccine, *TNFα* tumour necrosis factor alpha, *TT* tetanus toxoid, *VZV* varicella zoster virus

^a^These studies overlapped in patient population

The immunogenicity of the bivalent vaccine in 63 JIA patients was compared to 48 healthy controls, showing that all participants were seropositive up to 12 months after vaccination. GMCs were lower in patients than in controls, but no statistical significant difference in GMC over time was found [15].

Adequate immunogenicity of HPV vaccination is of specific interest in patients with systemic lupus erythematosus (SLE), as these patients have a high risk of persistent HPV infections [62–64]. Two studies included a total of 33 juvenile SLE (jSLE) patients, one including a control group of 49 healthy children. Both studies showed that the majority of patients seroconverted [16, 52]. Interestingly, the pilot study including six jSLE patients reported lower antibody concentrations in patients than in controls [16]. A study in 39 adult SLE patients also showed lower GMCs in patients than in healthy controls [65]. Based on these results, the long-term protection against HPV infections in SLE patients is unclear. Larger, controlled studies in jSLE patients are necessary to assess the immunogenicity of the HPV vaccine in this group.

#### Seasonal Influenza Virus and H1N1 Vaccine

Fifteen articles described the immunogenicity of seasonal influenza and H1N1 vaccines in pedRD (Table 2). They included 899 pedRD patients [23, 24, 27–29, 31, 32, 35, 36, 41–43, 56, 58]. One study group described a similar study population in three articles [31, 36, 58]. The three overlapping studies are described separately in Table 2, as it was impossible to disentangle the data.

Although antibody concentrations in patients were lower, seroprotection against influenza was similar in patients and controls. Two studies including 209 jSLE patients showed that this specific group has significantly lower seroconversion rates, seroprotection rates and GMT than healthy controls. Lower responses were not related to medications used and were possibly associated with a higher SLE disease activity index [32].

Two studies assessed the incidence of respiratory infections and influenza-like illness. Due to their small study population, no definite conclusions could be drawn regarding vaccine efficacy [23, 24].

#### Hepatitis A and hepatitis B Vaccine

One new study on the immunogenicity of the hepatitis A virus (HAV) vaccine was found [54]. Two studies including 57 patients showed an adequate immunogenicity in patients not using anti-TNFα treatment [40, 53]. One study in twelve children with inflammatory bowel syndrome using anti-TNFα treatment showed an adequate seroconversion rate of 92 % [54] (Table 2).

Hepatitis B virus (HBV) vaccines were studied in 245 patients [19, 21, 26, 30, 53] (Table 2). After vaccination, the majority of the patients and all of the healthy controls had protective antibody levels. However, the persistence of protective immunity against HBV may be lower in pedRD patients [19, 21]. The reduced proportion of patients that is protected directly after vaccination, together with the low percentage of protected patients several years after vaccination, illustrates that the humoral response after HBV vaccination should be checked and that patients could benefit from a booster vaccine.

#### Meningococcal Vaccine

One new study was found, in addition to the previously described study on the *Neisseria meningitides C* (NeisVac-C) vaccine, which was safe and immunogenic in 234 JIA patients [22, 59] (Table 2). In this study, MenC-IgG levels were assessed over time in 127 patients with JIA and 1527 healthy controls [22]. IgG levels decreased over time, with a faster decline in younger patients. Four years after vaccination, MenC-IgG levels in JIA patients were similar to those in healthy controls. Patients who had started biologicals showed an accelerated decline in antibody levels.

#### Pneumococcal Vaccines

No new studies were found on pneumococcal vaccines. In the previous review, one study was found (Table 2). It showed that JIA patients had a similar response and seroprotection rate to the 7-valent pneumococcal vaccine (PCV7) as healthy controls when using MTX or cyclosporine, either with or without concomitant GC use. Patients using anti-TNFα were all seroprotected, but had significantly lower antibody concentrations [39].

#### Tetanus-Diphtheria Vaccine

One new study was added to the evidence from four studies previously found on immunogenicity of the tetanus toxoid (TT) or tetanus-diphtheria (TD) vaccine [20, 55, 60, 61] (Table 2). The previously found studies (95 patients, 125 controls) showed comparable antibody levels to controls. Two studies in 430 pedRD patients assessed persistence of these antibodies over time. Both showed lower concentrations and seroprotection rates than in a comparable healthy control group after 7–16 years of follow-up [18, 20].

#### Other Non-live Composite Vaccines

No articles were found containing information on *Haemophilus influenza type B* (HiB) vaccines, pertussis vaccines or inactivated poliovirus vaccines. No information was found on vaccines indicated for endemic areas such as vaccines against typhoid fever, tick-borne encephalitis (FSME), rabies, Japanese encephalitis or cholera.

### Live-Attenuated Vaccines

#### Measles, Mumps and Rubella Vaccine

In the previous systematic literature review, only one study in ten JIA patients assessed short-term immunogenicity of the MMR booster. It showed a cellular and humoral immune response comparable to healthy controls [34]. One additional article on the immunogenicity of the MMR booster vaccination was published. This RCT showed that all 68 vaccinated patients displayed a significant increase in MMR antibody concentrations. All patients were seroprotected against MMR at 12 months after vaccination [14••] (Table 2).

Two studies reported on the persistence of antibodies several years after MMR vaccination in patients with JIA or jSLE. Both studies found similar levels of protective antibodies against measles in patients and controls 7–16 years after two MMR doses in the first year of life [20] and in all age groups (1–19 years) after one or two MMR doses. Protective antibody levels against mumps and rubella up to 10 years after MMR booster vaccination were significantly lower in JIA patients than in controls. Patients had an odds ratio of 0.4 to be seroprotected against mumps or rubella compared to controls (adjusted for age and number of vaccinations) [18] (Table 2).

#### Varicella Zoster Vaccine

In the previous review, two studies regarding the varicella zoster virus (VZV) vaccine were found. A controlled study including 25 pedRD patients and 18 healthy controls found a lower response rate in patients than in controls after vaccination. Of the eight patients who reported having contact with a VZV-infected individual, two (both non-responders), developed chickenpox [17]. A case series reported six IBD patients having positive immunity after vaccination [38]. One new study, an RCT including 54 jSLE patients of whom 28 were vaccinated, has been found in the new search. Only patients who used either cyclosporine, azathioprine, methotrexate and/or GC up to 20 mg/day were included in this study. All participants had protective antibody levels against VZV at baseline. Patients had a similar increase in GMT as the healthy control group, and all had a significant increase in antibody levels compared to baseline. Over 35.6 months of follow-up after vaccination, four cases of HZ were reported in the unvaccinated group whereas no HZ occurred in the vaccinated group [13••] (Table 2).

In adults, two large studies illustrate the importance of effective vaccination against VZV. A meta-analysis in adults with rheumatic diseases (RD) showed that the risk of HZ infections is increased by up to 61 % in patients using biologicals compared to patients using conventional disease-modifying anti-rheumatic drugs (DMARDs) [66]. A retrospective cohort study in 7780 vaccinated and 455,761 unvaccinated adults with RD assessed vaccine efficacy (incidence of HZ infections >42 days after vaccination). They showed a significantly lower hazard ratio for HZ infections (HR 0.61, 95 % CI 0.59–0.75) in vaccinated patients up to 2 years of follow-up [67•].

#### Bacillus Calmette-Guérin Vaccine

No new evidence was found on the immunogenicity of the Bacillus Calmette-Guérin (BCG) vaccine in pedRD patients [27, 44–49] (Table 2). In the 2011 review, seven papers were described including 15,810 Kawasaki disease (KD) patients and 115 JIA patients. It is suggested that JIA patients have lower protection rates after vaccination, due to their lower tuberculin skin test induration size. The remaining articles did not assess immunogenicity. As the vaccine causes local inflammation at the BCG vaccination site in up to 50 % of KD patients, withholding the BCG vaccine in active KD was advised [11••].

#### Yellow Fever Vaccine

No studies were found on the immunogenicity of the yellow fever (YF) vaccine in children with pedRD, but it has been studied in 91 adult patients with RD. In these patients, the vaccine had good immunogenicity. The responses were reduced in the 26 patients who used anti-TNFα therapy [71]. The EULAR stated that booster vaccinations against YF can be considered in patients on MTX less than 15 mg/m^2^/week or low-dose GC [12••].

## Vaccine Safety in Paediatric Patients With Rheumatic Diseases

### Adverse Events and Serious Adverse Events

Adverse events (AE) and serious adverse events (SAE) were registered in the majority of the studies. None found relevant differences in AE between patients and controls and no SAE related to vaccination were reported.

Preliminary data on thromboembolic events after qHPV vaccination resulted in the EULAR recommendation to be vigilant for these complications [11••]. Based on current literature, this seems unnecessary as a large cohort study in 997,585 healthy girls, of whom 296,826 received at least one dose of the qHPV vaccine, showed no evidence of an association between qHPV vaccination and venous thromboembolic adverse events [68•].

### Disease Activity Induced by Vaccination

As most pedRD are very unpredictable in disease activity and flares, the only reliable method to assess the effect of vaccination on disease activity is an RCT study design. This way, results are corrected for the relapsing-remitting course of the disease. Two RCTs assessed the effect of the live-attenuated MMR booster vaccination on JIA, respectively, the VZV vaccination on jSLE disease activity. Both studies reported similar disease activity and flare rates in vaccinated patients and disease-controls [13••, 14••]. Some non-randomized studies included an unvaccinated control group of patients. These studies suffered from selection bias since the unvaccinated group had lower disease activity at baseline [24] or the control group was not described at all [30].

Most studies assessing disease activity used patients as their own control. These studies reported a stable disease activity over time or similar flare rates before and after vaccination (Table 2). One case report described a systemic onset JIA (soJIA) patient on anti-IL6 who received two seasonal influenza vaccines and had a disease flare after both vaccinations [57]. In contrast, a study in 27 soJIA patients using anti-IL6 receiving a seasonal influenza vaccine did not show any exacerbations [42]. In summary, studies do not show an increase in disease activity after vaccinations in the patient population as a whole. This is unequivocally shown by the RCTs with live-attenuated vaccines. Of course, the theoretical possibility remains that individual patients are susceptible for aggravation of disease after vaccination due to disproportionate immune responses. However, this theoretic possibility should not result in refraining from current immunization practice, because the benefits of infection prevention significantly outweigh the small risk of a disease flare in patients.

### Induction of Infections With Attenuated Pathogens

The possibility of the induction of infections with an attenuated pathogens after live-attenuated vaccines is a matter of concern especially in patients on high-dose immunosuppressive drugs or biologicals. Only the RCT assessing the safety of the live-attenuated MMR vaccine included patients using biologicals (*n* = 9). They did not have infections with the live-attenuated pathogens [14••]. A cohort study including 25 VZV-vaccinated pedRD patients showed no overt varicella episodes within 40 days after vaccination. Three patients did develop a mild, self-limiting varicella-like rash, but this was not accompanied by any other symptoms [17]. A study in 7780 adult patients with RD reported 11 HZ cases within 42 days after vaccination, suggestive for vaccination-induced herpes infections [67•]. Reassuringly, no vaccination-induced HZ infections were detected in the 633 patients on biologicals.

Information on the BCG vaccination in patients on high-dose immunosuppressive drugs or biologicals is lacking. There is a very high rate of complications in patients who are severely immunocompromised, such as SCID patients [69]. One case report described a 3-month-old infant born to a mother with Crohn’s diseases using infliximab who had a lethal vaccination-induced mycobacterial infection after BCG vaccination [70]. Based on these data, BCG vaccines should therefore not be administered to patients using biologicals or high doses of immunosuppression.

There is no information available on the safety of the live-attenuated YF vaccine in patients with pedRD, but the vaccine was safe in adult patients with RD [71].

## Adjuvant Safety in Paediatric Patients With Rheumatic Diseases

Adjuvants are added to vaccines to enhance the immune response to the vaccine-antigen. Frequently used adjuvants (alum, Toll-like receptor (TLR) four ligand monophosphoryl lipid A adsorbed to alum (ASO4) and oil in water emulsions like AS03 or MF59) stimulate pattern-recognition receptors (PRRs) such as TLRs. TLRs are expressed on cells like dendritic cells, which in turn determine the magnitude and quality of the adaptive immune response [72–74]. Through these mechanisms, adjuvants could theoretically also trigger or enhance autoimmune responses in patients with established AID [74, 75].

The safety of adjuvants in rheumatic diseases has not been studied well. RCTs, in which patients with rheumatic diseases are vaccinated with adjuvanted versus unadjuvanted vaccines are lacking. In this review, we found 12 reports including 614 patients that studied an adjuvanted non-live vaccine [15, 16, 21, 26, 30, 35, 39, 41, 52–54, 59]. Seven reports including 499 patients studied a non-adjuvanted non-live vaccine [24, 25, 31, 32, 36, 56, 58]. It was unclear whether the vaccine was adjuvanted in eight reports [19, 22, 23, 40, 42, 55, 57, 61]. No marked increase in disease activity was seen in the patients receiving an adjuvanted vaccine compared to the patients who received an unadjuvanted dead composite vaccine. Based on these results, it does not seem likely that adjuvants cause a significant deterioration of disease activity in paediatric patients with rheumatic diseases.

In theory, adjuvants could also be part of the causal pathway in the onset of AID. Anecdotal evidence for this relation has been published [75, 76], and a syndrome of shared clinical symptoms thought to be caused by adjuvants, the autoimmune/inflammatory syndrome induced by adjuvants (ASIA), has been postulated [76]. Recently, a large epidemiological study applying the ASIA diagnostic criteria to a population vaccinated with the HPV vaccine has been performed. A total of 57 million administrated doses were reported and 26,508 self-reports on AEs were found. Of these, 3932 cases could be classified as ASIA, defined by flu-like symptoms such as fever, myalgia, arthralgia or arthritis. In 2634 cases, a probable or possible association with HPV vaccination could be made. However, no mention was made about the duration of the complaints, and a comparison of the frequency of similar complaints in an unvaccinated population was not made [77].

Several large studies have not found any association between vaccination and AID. A large register-based cohort study including 997,585 girls aged 10–17 years, among whom 296,826 received a total of 696,420 qHPV vaccine doses, no association between exposure to the qHPV vaccine and autoimmune adverse events was found [68•]. Also, analysis of over 68,000 participants who received AS04-adjuvanted vaccines or served as controls demonstrated a low rate of autoimmune disorders, without evidence of an increase in relative risk associated with AS04-adjuvanted vaccines [78]. Finally, a review of reported adverse reactions after the pandemic influenza A/H1N1 vaccine using EudraVigilance data and literature did not reveal a difference between autoimmune phenomena after adjuvanted or non-adjuvanted A/H1N1 vaccines [79]. Thus, the possible relation between vaccine adjuvants and the induction of autoimmune rheumatic diseases is thus far not substantiated.

## Discussion

The current systematic literature review found 21 articles on vaccinations in pedRD published since the last systematic literature from 2011. The new evidence, selected using the same criteria as the first review, was added to the 27 previously described studies [12••].

Vaccines are generally immunogenic in patients with pedRD. The validity of available evidence for the effect of immunosuppressive drugs on immunogenicity was moderate or low. To accurately assess the effect of a drug on the immunogenicity of a vaccine, patients using these drugs need to be compared to patients who are drug-free or are using a minimal amount of immunosuppression. Few studies included such a comparison, so only indirect conclusions on the effects of GC, MTX and biologicals could be drawn. GC, predominantly studied in a low dose (<20 mg/day), and MTX do not have detrimental effects on the immune response. More evidence has become available on the effect of biologicals, especially anti-TNFα treatment, on (long-term) immunogenicity of vaccines. Although seroprotection rates are usually adequate, antibody concentrations are lower in patients using biologicals.

To ensure long-term protection against vaccine-preventable infections, protective antibody levels should be persistent. Persistence of protective antibody levels is lower in pedRD patients than healthy controls for some, but not all, pathogens [18, 22]. Biological use seems to accelerate the natural decline of antibody levels, besides lowering the vaccine-induced antibody concentrations. Therefore, regular assessment of antibody levels and subsequent administration of booster vaccines in these patients is important to ensure long-term protection. Studies in healthy individuals suggest that circulating antibody levels alone may not be predictive of long-term protection, as cellular immunity can persist independent of antibody levels [80, 81]. Assessment of cellular memory in vaccinated pedRD patients could help to study long-term protection against vaccine-preventable diseases.

Evidence on the efficacy (i.e. infection prevention) of vaccines in pedRD is still lacking. The studies that measured infection rates in vaccinated and unvaccinated patients were underpowered for definite conclusions on efficacy.

Regarding safety, vaccinations do not cause serious adverse events. Disease activity is not influenced by vaccination in the majority of the patients, now unequivocally shown for the MMR vaccination in JIA patients and the VZV vaccination in jSLE patients. No evidence has been found that adjuvants cause a higher disease activity in pedRD.

No vaccine-induced infections with live-attenuated viruses were reported in vaccinated JIA or jSLE patients after the MMR and VZV booster vaccination, respectively. Therefore, it seems that these booster vaccinations can be administered to pedRD patients, even in patients using biologicals. BCG vaccinations should not be administered to patients on high-dose immunosuppressive drugs or biologicals due to lack of safety data. Larger, controlled studies are necessary to study rare serious adverse events, especially in patients on high-dose immunosuppressive drugs or biologicals.

Much information on vaccination in pedRD has been gained in the time since the initial systematic review. For some vaccines, high-quality studies have been performed that show that they are generally immunogenic and safe. Additionally, the need for (booster) vaccinations in pedRD has been illustrated by the papers published on reduced persistence of immunological memory over time.

More evidence on the influence of biologicals on the immune response and safety of vaccines is required. Although we have information on the immunogenicity of many vaccines, this does remain a surrogate endpoint. The efficacy, namely a decrease in infection rates in pedRD, needs to be studied in larger cohorts.

While more information will be gathered over the coming years, we can now conclude that vaccinations in pedRD should be advocated. Paediatric rheumatologists should be pro-active in assessing protective antibody levels in pedRD patients and should, in line with the EULAR-recommendations, administer booster vaccines to children who are not adequately protected.
